# Lysis of a Lactococcus lactis Dipeptidase Mutant and Rescue by Mutation in the Pleiotropic Regulator CodY

**DOI:** 10.1128/AEM.02937-19

**Published:** 2020-04-01

**Authors:** Chenxi Huang, Jhonatan A. Hernandez-Valdes, Oscar P. Kuipers, Jan Kok

**Affiliations:** a Department of Molecular Genetics, University of Groningen, Groningen Biomolecular Sciences and Biotechnology Institute, Groningen, the Netherlands; University of Helsinki

**Keywords:** *Lactococcus lactis*, dipeptidase PepV, global repressor CodY, peptidoglycan synthesis, intracellular alanine, nitrogen metabolism, cell morphology

## Abstract

Lactococcus lactis subsp. *cremoris* MG1363 is a model for the lactic acid bacteria (LAB) used in the dairy industry. The proteolytic system, consisting of a proteinase, several peptide and amino acid uptake systems, and a host of intracellular peptidases, plays a vital role in nitrogen metabolism and is of eminent importance for flavor formation in dairy products. The dipeptidase PepV functions in the last stages of proteolysis. A link between nitrogen metabolism and peptidoglycan (PG) biosynthesis was underlined by the finding that deletion of the dipeptidase gene *pepV* (creating strain MGΔ*pepV*) resulted in a prolonged lag phase when the mutant strain was grown with a high concentration of glycine. In addition, most MGΔ*pepV* cells lyse and have serious defects in their shape. This phenotype is due to a shortage of alanine, since adding alanine can rescue the growth and shape defects. Strain MGΔ*pepV* is more resistant to vancomycin, an antibiotic targeting peptidoglycan d-Ala–d-Ala ends, which confirmed that MGΔ*pepV* has an abnormal PG composition. A mutant of MGΔ*pepV* was obtained in which growth inhibition and cell shape defects were alleviated. Genome sequencing showed that this mutant has a single point mutation in the *codY* gene, resulting in an arginine residue at position 218 in the DNA-binding motif of CodY being replaced by a cysteine residue. Thus, this strain was named MGΔ*pepVcodY*^R218C^. Transcriptome sequencing (RNA-seq) data revealed a dramatic derepression in peptide uptake and amino acid utilization in MGΔ*pepVcodY*^R218C^. A model of the connections among PepV activity, CodY regulation, and PG synthesis of L. lactis is proposed.

**IMPORTANCE** Precise control of peptidoglycan synthesis is essential in Gram-positive bacteria for maintaining cell shape and integrity as well as resisting stresses. Although neither the dipeptidase PepV nor alanine is essential for L. lactis MG1363, adequate availability of either ensures proper cell wall synthesis. We broaden the knowledge about the dipeptidase PepV, which acts as a linker between nitrogen metabolism and cell wall synthesis in L. lactis.

## Abstract (precis)

Precise control of peptidoglycan synthesis is essential in Gram-positive bacteria for maintaining cell shape and integrity as well as resisting stresses. Although neither the dipeptidase PepV nor alanine is essential for L. lactis MG1363, adequate availability of either ensures proper cell wall synthesis. We broaden the knowledge about the dipeptidase PepV, which acts as a linker between nitrogen metabolism and cell wall synthesis in L. lactis.

## INTRODUCTION

Lactococcus lactis is a Gram-positive bacterium and, due to its importance in dairy industry, one of the best-studied lactic acid bacteria (LAB) (1). L. lactis MG1363 is a plasmid-free laboratory model strain of which, among many other attributes, the proteolytic system was well elucidated decades ago. Understanding milk protein breakdown by L. lactis can help industry to change the flavor profile of dairy products (2). The genes of the major proteinase, all peptidases, several peptide uptake systems, and the global transcriptional regulator of nitrogen and carbon metabolism have all been cloned and examined in great detail by gene overexpression and knockout studies (3–6).

Milk fermentation by L. lactis involves multiple stages of casein degradation: the cell envelope-associated proteinase (PrtP) degrades casein into (oligo)peptides, which are transported by the oligopeptide transport system (Opp) and di- and tripeptide transport systems (DtpT, and Dpp). After entry into the cytoplasm, the peptides are degraded by an array of different peptidases into amino acids for further utilization (4). One of these is the dipeptidase PepV, which is responsible for degrading a wide range of dipeptides; the enzyme is conserved in LAB such as Lactobacillus delbrueckii subsp. *bulgaricus*, Lactobacillus helveticus SBT 2171, and Lactobacillus sakei (7).

Our previous work has shown that deletion of the dipeptidase gene *pepV* has no effect on the growth rate in the rich M17 medium, but significantly slower growth was observed when the mutant was grown in milk (8). Peptides are the major nitrogen source in M17, while in milk it is intact casein. This implies that during dairy fermentation PepV might play an important role in liberating certain amino acids that affect the growth rate.

**FIG 1:**
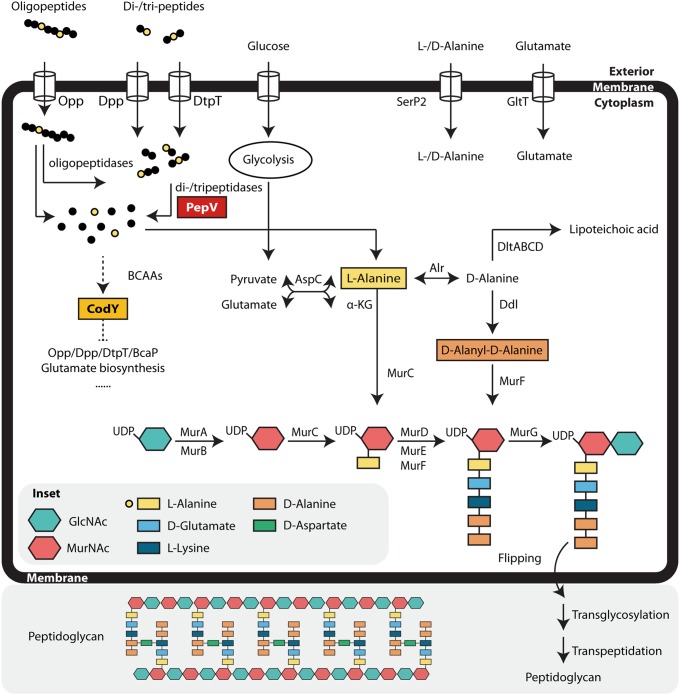
Metabolic pathways relevant to intracellular alanine pool in L. lactis MG1363. Partial pathways were adapted from KEGG (www.genome.jp/kegg/pathway.html). Oligopeptide permease (Opp) internalizes oligopeptides containing 4 to 35 amino acid residues, and dipeptide permease (Dpp) and ion-linked di- and tripeptide transporter (DtpT) take up di- and tripeptides (3). A host of different intracellular amino- and endopeptidases, among which is the dipeptidase PepV, degrade peptides into smaller peptides and, ultimately, amino acids (4). Glycolysis, e.g., using glucose, contributes to pyruvate formation (38, 39). dl-Alanine/dl-serine/glycine transporter (SerP2) imports extracellular alanine (10). ATP-driven glutamate/glutamine transporter (GlnPQ) (40) and aspartate/glutamate transporter (AcaP) import glutamate (41). Branched-chain amino acids (BCAAs; Leu, Ile, and Val) are corepressors of the pleiotropic transcriptional regulator CodY, which represses nitrogen metabolism (22). Alanine-synthesizing transaminase (AspC) converts pyruvate and glutamate into alanine and α-ketoglutarate (11). Alanine racemase (Alr) catalyzes interconversion of l-Ala and d-Ala. DltABCD are involved in d-alanylation of lipoteichoic acid (LTA) (31); Ddl ligates d-Ala to d-Ala–d-Ala (42). MurABCDEFG catalyze peptidoglycan (PG) precursor synthesis, which through the indicated additional enzymatic steps leads to the formation of the mature PG (9).

Alanine is not essential to L. lactis MG1363 since it can be synthesized by the organism, but it is important in peptidoglycan (PG) synthesis (9). Thus, a sufficiently large intracellular alanine pool is vital for proper cell growth. Multiple processes in L. lactis MG1363 contribute to the pool of intracellular alanine: the uptake of oligopeptides and di- and tripeptides containing alanine residues through the Opp, Dpp, and DtpT transporters mentioned above, the further degradation by peptidases to liberate the alanine (4), the uptake of free alanine from the medium via the dl-Ala transporter SerP2 (10), and alanine synthesis from pyruvate and glutamate by the transaminase AspC (11) (Fig. 1).

Amino acid metabolism in bacteria is normally regulated by biochemical control of specific enzymes or response to certain metabolites. However, more global regulation also exists at the level of gene transcription. CodY is a pleiotropic repressor that is well conserved in low-GC-containing Gram-positive bacterial species. It was first identified in Bacillus subtilis as a repressor of the dipeptide transport (*dpp*) operon (12). Later studies showed that CodY more generally controls nitrogen metabolism, while in some pathogens it also regulates virulence gene expression (13). Previous studies from our laboratory and others have identified the regulon of L. lactis CodY (14, 15). The majority of the CodY-dependent genes in L. lactis are involved in the degradation of casein, peptide and amino acid transport, and metabolism. The major peptide uptake systems in a CodY deletion strain are highly upregulated compared to those in the wild-type L. lactis MG1363, which might severely alter the intracellular nitrogen pool (14).

In this study, we observed that of 14 peptidase knockout mutants of L. lactis, only the dipeptidase PepV mutant did not grow in the presence of a high concentration of glycine. We show that dipeptidase PepV affects PG synthesis by influencing the intracellular alanine pool. A *pepV* knockout mutant has a severely prolonged lag phase in the presence of glycine in the medium, with cells showing defects in their shape and separation ability. After cultivation of the *pepV* knockout mutant, a new mutant with a shorter lag phase was obtained. Genome and RNA sequencing revealed that this shorter lag phase was affected by a point mutation in the global repressor *codY*, resulting in the derepression of the corresponding CodY regulon.

## RESULTS

### Dipeptidase PepV affects growth and cell morphology.

M17 media with glucose (GM17) supplemented with glycine (1.5%, wt/vol) and sucrose (0.5 M) (SMGG) are widely used for making competent cells of L. lactis. The principle is that glycine weakens the cell wall, while sucrose works as an osmotic pressure stabilizer (16).

**FIG 2:**
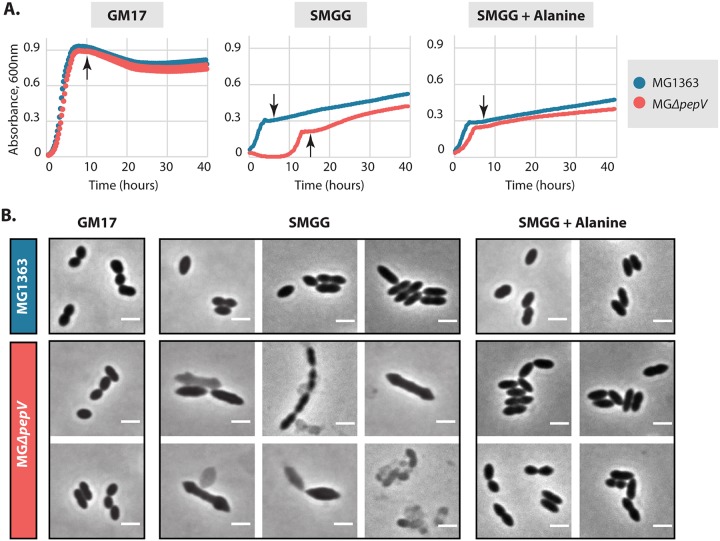
Growth and morphology changes in MG*ΔpepV*. (A) Growth at 30°C of L. lactis MG1363 and its isogenic mutant MG*ΔpepV* in GM17, SMGG, and SMGG plus alanine. Curves are the means of triplicates. (B) Light microscopy images of MG1363 and MG*ΔpepV* under conditions corresponding to those for panel A. Samples were taken after cultures reaching stationary phase (arrows in panel A). Typical examples are shown. White bars, 2 μm.

Interestingly, we observed during the construction of multiple peptidase mutants of L. lactis that an L. lactis MG1363 derivative lacking the dipeptidase gene *pepV* (strain MGΔ*pepV*) does not grow overnight in SMGG when inoculated directly from a glycerol stock. None of the other 14 peptidase knockout mutants had that problem (data not shown). However, when the glycerol stock was first grown overnight in GM17 and then inoculated in SMGG, an increase in culture optical density (OD) at 600 nm of L. lactis MGΔ*pepV* was consistently observed after around 15 h (Fig. 2A). Light microscopy analysis of MGΔ*pepV* in SMGG media revealed several morphological changes in a large fraction of the cells compared to the control strain MG1363 under the same conditions (Fig. 2B). While the wild-type strain showed normal lactococcal morphology in both GM17 and SMGG media, many of the MGΔ*pepV* cells in SMGG had pointed ends, a larger cell size, or a grayish color with a blurred border indicative of cell lysis. In addition, long chains of cells were also observed.

Previously, it was shown that L. lactis dipeptidase PepV can liberate alanine from dipeptides (8). Hammes et al. (17) have shown that a high concentration of glycine disrupts peptidoglycan (PG) biosynthesis in several species of Gram-positive bacteria, as it replaces the alanine residues in the PG precursor. Although L. lactis contains several other (amino)peptidases that could also liberate alanine from peptides available in the rich SMGG medium, their activities are, apparently, not enough to compensate for the PepV deficiency, nor is the concentration of free alanine. To determine whether the prolonged lag phase in the growth of MGΔ*pepV* might be caused by a lack of alanine, we added alanine to SMGG. Indeed, the growth in SMGG could be restored to almost wild-type levels by this addition, while the morphology of MGΔ*pepV* was also recovered (Fig. 2). These observations indicate that a relationship exists between PepV and PG synthesis. To exclude an effect of other amino acids, we added each of 17 other amino acids, but none of them restored growth of MGΔ*pepV* in SMGG (see Fig. S1 in the supplemental material). As the dipeptide Ala-Ala allowed MGΔ*pepV* to grow normally in SMGG, there are apparently not enough alanine-containing peptides in GM17 medium to fulfill the alanine requirement (Fig. S1). We therefore hypothesize that PepV affects PG synthesis by contributing to the intracellular alanine pool and that none of the other (amino)peptidases can fully take over that function.

### L. lactis MG*ΔpepV* is more resistant to vancomycin than the wild type.

**FIG 3:**
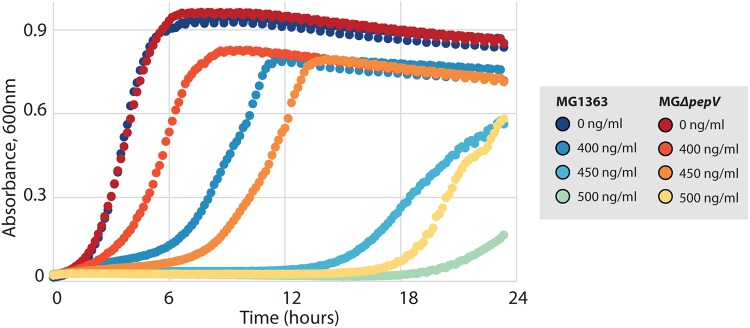
MG*ΔpepV* is more resistant to vancomycin. Colors from dark to light represent vancomycin concentrations of 0 ng/ml, 400 ng/ml, 450 ng/ml, and 500 ng/ml as shown. Growth curves represent means of triplicates.

Vancomycin inhibits cell wall synthesis by binding to the d-Ala–d-Ala terminal of the growing peptide chain during cell wall synthesis. It has been reported that after d-Ala is replaced with d-Lac in the pentapeptide of the PG precursor, L. lactis becomes more resistant to vancomycin and shows defects in cell elongation and separation (18). A vancomycin resistance test was performed by inoculating L. lactis MG1363 and MGΔ*pepV* in GM17 with different concentrations of vancomycin. MGΔ*pepV* is more resistant to vancomycin than its parent (Fig. 3). This suggests that the d-Ala in the PG precursor of MGΔ*pepV* has been replaced, indicating that PepV is a major contributor to the intracellular alanine pool.

### L. lactis MG*ΔpepV* dies fast in SMGG and regrows after a long lag phase.

**FIG 4:**
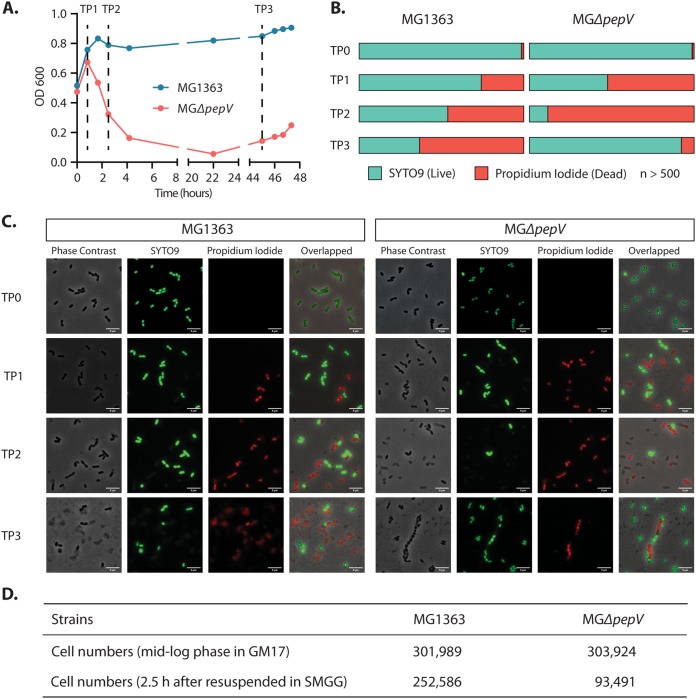
Viability of L. lactis MG*ΔpepV*. Four time points were analyzed. At TP0 (not indicated in panel A), a sample of the cells grown in GM17 until mid-log phase was taken just prior to the start of the experiment where cultures were spun down, washed, and resuspended in SMGG and further incubated at 30°C. TP1, TP2, TP3, 50 min, 2.5 h, and 45 h after resuspension in SMGG, respectively. (A) Growth curves of L. lactis MG1363 and MG*ΔpepV* growing in SMGG. (B) Percentage of live/dead cells of L. lactis MG1363 and MG*ΔpepV* at each time point; in all cases, more than 500 cells were counted. (C) Light microscopy images of cultures of MG1363 and MG*ΔpepV* at each time point. Typical examples are shown. White bars, 5 μm. (D) MG1363 and MG*ΔpepV* cells were grown in GM17 until mid-log phase, sampled (5 μl each), and then transferred to SMGG for 2.5 h and sampled (5 μl each). All 4 samples were analyzed by flow cytometry to calculate the numbers of cells.

Because of the large differences in growth behavior in the presence of glycine, it is very hard to compare the two strains MG1363 and MG*ΔpepV* in similar growth stages at the same time. Also, it is impossible to examine the intermediate and direct responses of the MG*ΔpepV* strain to glycine since it needs some 10 h for visible growth to be seen. To circumvent these problems, the cells were first inoculated into GM17 to allow them to grow, and when the cultures reached the log phase, the cells were spun down and resuspended in SMGG. As is clear from Fig. 4A, the growth curve of L. lactis MG*ΔpepV* was quite different from that of MG1363. The OD of the MG*ΔpepV* culture first slightly increased, after which it decreased sharply within 3 h. Subsequently, after a very long lag phase (around 40 h), the OD started to increase again.

In addition to examining the growth characteristics of the two strains, we also sampled the cultures at 4 time points (TP), namely, in the log phase in GM17 just prior to inoculation in SMGG (TP0) and at 50 min (TP1), 2.5 h (TP2), and 45 h (TP3) in SMGG. The last two time points correspond with the early and late stationary phases of MG1363 and the lysis phase and the start of the regrowth of MGΔ*pepV* (Fig. 4A). The cells were subjected to LIVE/DEAD cell staining in order to observe cell status and any morphological changes. As shown in Fig. 4B, the percentage of living L. lactis MGΔ*pepV* cells corresponded to the OD change in Fig. 4A. In the culture of MGΔ*pepV* at TP2, dead cells were observable in the form of grayish cells that were stained by propidium iodide. Clearly, significantly more of those dead cells are present in the MGΔ*pepV* culture than in that of MG1363. At TP3, while the MG1363 culture contains a majority of dead cells, the MGΔ*pepV* mutant started to regrow (Fig. 4B). Cell lysis of MGΔ*pepV* was indicated by the optical density decrease and by the results of a flow cytometry analysis (Fig. 4D). A constant culture volume (5 μl) was analyzed to calculate the number of cells for each strain. Samples were taken at the mid-log phase in GM17 and 2.5 h after the strains were transferred into SMGG. These time points correspond to TP0 and TP2 in the LIVE/DEAD staining experiment. The cell number dropped considerably more in the MGΔ*pepV* culture after 2.5 h in SMGG.

### Cultivation of L. lactis MGΔ*pepV* in SMGG leads to faster restoration of growth.

When we inoculated MG*ΔpepV* into SMGG medium in a 96-well microtiter plate, growth in all of the wells was ultimately observed, with some of the cultures showing shorter lag phases. We reinoculated cells from those “faster” cultures in fresh SMGG repeatedly and consecutively. After 10 days of repeated cultivation in SMGG, a single colony isolate was obtained that grew faster in SMGG than MG*ΔpepV*. We have labeled this strain MGΔpepV*. To determine whether the phenotype of MGΔpepV* was caused by adaptation or mutation(s), the strain was grown overnight in GM17 and consecutively grown and reinoculated in GM17 daily for 10 days (approximately 150 generations). As no deterioration of growth was observed, the reversal of the phenotype was most probably caused by one or more stable mutations (see below).

**FIG 5:**
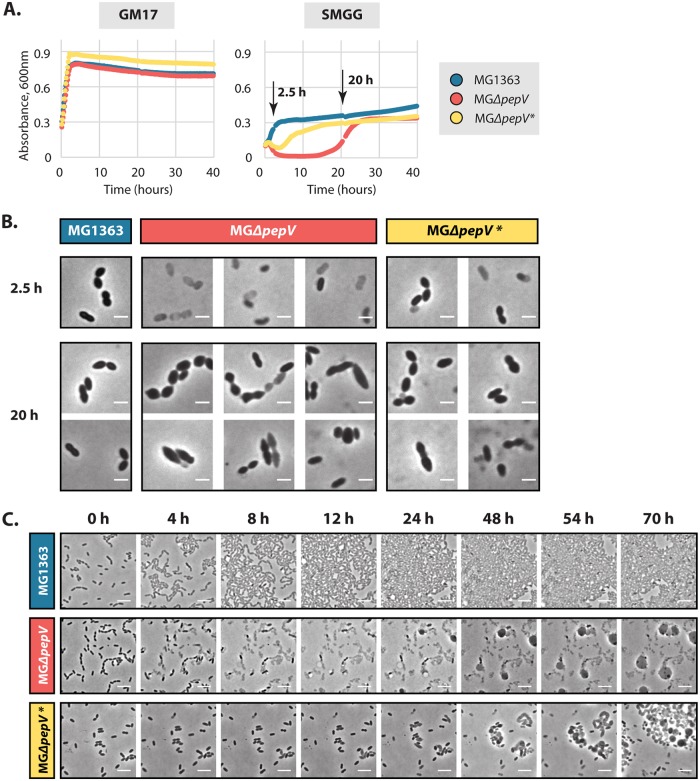
L. lactis MGΔpepV* has a short lag phase in SMGG. (A) Growth at 30°C of L. lactis MG1363, MG*ΔpepV*, and MGΔpepV* in GM17 and SMGG. Growth curves are the means of triplicates. Arrows, time points for analysis in panel B. (B) Microscopy images of MG1363, MG*ΔpepV*, and MGΔpepV* grown in SMGG. Samples were taken at 2.5 h and 20 h after resuspension in SMGG (arrows in panel A). White bars, 2 μm. (C) Time-lapse microscopy snapshots of MG1363, MG*ΔpepV*, and MGΔpepV* grown at 30°C on a microscopy slide carrying a thin slab of SMGG agar. White bars, 5 μm. Snapshots were taken from Movies S1 to S3. In panels B and C, typical examples are shown.

In addition to examining the growth characteristics of the strains, we inoculated three strains (MG1363, MGΔ*pepV*, and MGΔpepV*) in GM17. When the cultures reached the log phase, the cells were spun down and resuspended in SMGG (Fig. 5A). In order to observe any morphological changes under the microscope, we also sampled the cultures at different time points (2.5 h and 20 h in SMGG). Some ghost-like cells were observed in the cultures of both mutants after 2.5 h in SMGG (Fig. 5B). Clearly, in the culture of MGΔ*pepV* more of those ghost cells were present than in that of MGΔpepV*. After 20 h, similar morphological changes were seen for MGΔ*pepV*, as presented above (compare Fig. 2B with Fig. 5B; cells in long chains, cells with pointed ends, and grayish cells). All of these changes are indicative of MGΔ*pepV* having problems in cell wall synthesis and/or cell separation. As for MGΔpepV*, the morphology was in between that of the wild type and MGΔ*pepV* (Fig. 5B).

Since we only monitored the regrowth of MGΔ*pepV*, and not of MGΔpepV*, we then performed time-lapse microscopy on SMGG agar slides in order to visualize the restoration of growth of MGΔpepV*. The growth patterns of the three strains on the SMGG agar slides are consistent with those of the liquid cultures. The wild-type MG1363 cells grew and divided as normal, while most of the MG*ΔpepV* cells lysed within 12 h, and many fewer MGΔpepV* cells lysed within the same time frame. After 40 h, regrowth of only the MGΔpepV* cells was observed, but the cells were longer and did not separate smoothly. As for MG*ΔpepV*, regrowth was not observed during the entire analysis period (10 days). After 24 h, some “ballooning” was taking place and disappearing in the MG*ΔpepV* samples, a phenomenon that was not seen in the cultures of the other two strains. These structures are probably caused by fusion of membranous material after the cells have lysed, since the bubbles appeared not randomly on the slide but always in the vicinity of lysing cells. Upon disintegration of the bubbles, the remainder seems to stick to the cover slide, making it less likely that they were gas bubbles (Fig. 5C and Movies S1 to S3).

### Genome sequencing shows that MGΔpepV* carries a single mutation, specifying CodY^R218C^.

Based on the observations presented above, we decided to sequence the genomes of MGΔ*pepV* and MGΔpepV*. Only one point mutation (CGT→TGT) was present in MGΔpepV* relative to its parent, MGΔ*pepV*, leading to a change of amino acid residue 218 of the pleiotropic repressor protein CodY. The single base change replaced the charged arginine residue at that position in CodY with a noncharged cysteine residue, so we renamed L. lactis MGΔpepV* as L. lactis MG*ΔpepVcodY*^R218C^, which is used through the rest of this article. Yuan et al. (19), in their molecular docking and molecular dynamics simulations study, predicted that L. lactis CodY Arg^218^ plays a vital role in DNA binding of the protein. When B. subtilis CodY arginine residue 214 (Arg^214^, corresponding to Arg^218^ of L. lactis) was changed into a glutamate residue, CodY DNA binding ability was strongly affected (20). Thus, possible DNA binding defects of the mutated CodY repressor in MG*ΔpepVcodY*^R218C^ might explain why it regrows much faster than MG*ΔpepV*. To verify this hypothesis, a transcriptomic experiment was performed.

### The CodY regulon is highly upregulated in L. lactis MG*ΔpepVcodY*^R218C^ in SMGG.

To assess the effects of glycine on gene expression in MG*ΔpepV* and to explore how CodY^R218C^ helped rescue MGΔ*pepVcodY*^R218C^, their transcriptomes were compared with that of L. lactis MG1363. The strains were cultured in GM17 until the mid-log phase (OD = 0.7) was reached, after which the cells were spun down, washed, and resuspended in SMGG. The cells were further incubated for 30 min, after which total RNA was isolated. This setup was chosen to ensure that the cells were affected by glycine and the transcripts were isolated before any major cell lysis would occur. The data of two comparisons were analyzed, MG*ΔpepV* versus MG1363 and MG*ΔpepVcodY*^R218^ versus MG1363, using the T-Rex software (21). For context simplicity, the names MG*ΔpepV* and MG*ΔpepVcodY*^R218^ are used in this section, corresponding to the two comparisons, respectively.

**FIG 6:**
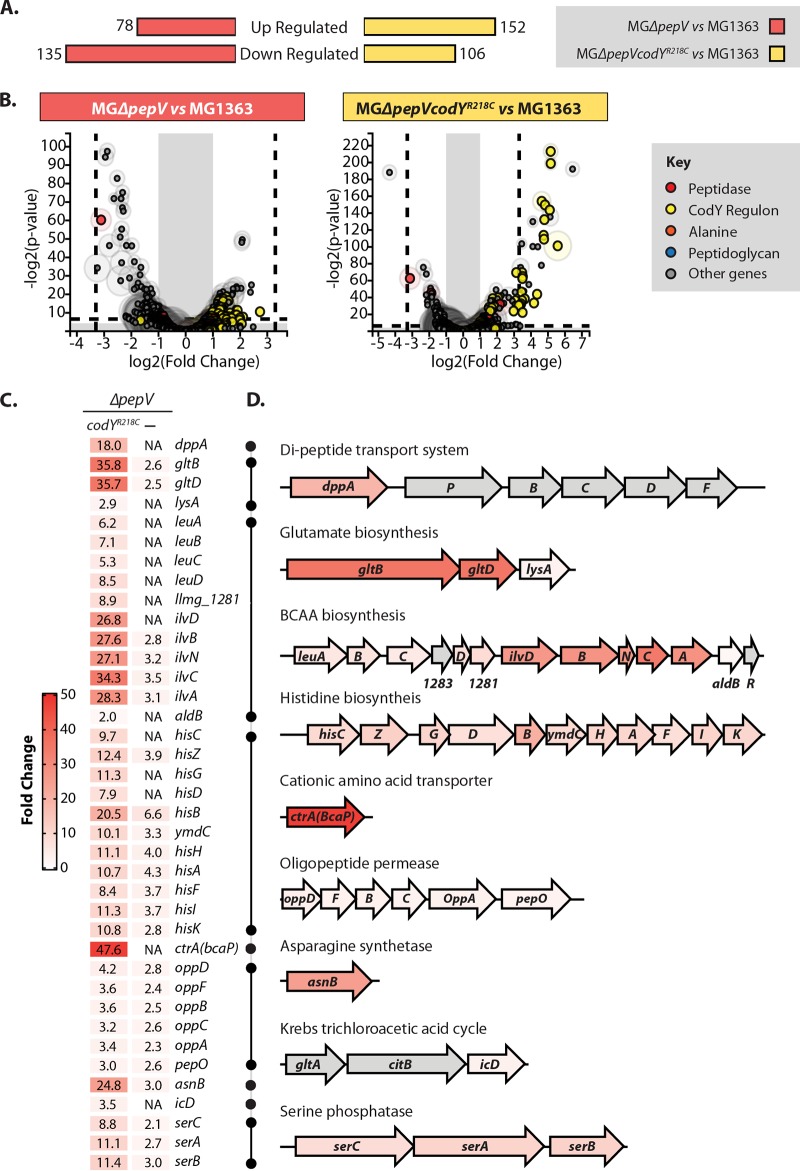
RNA-seq confirms upregulation of CodY regulon. (A) Absolute number of genes that are upregulated or downregulated in MG*ΔpepV* or MG*ΔpepVcodY*^R218C^ in comparison with MG1363. (B) T-Rex-generated volcano plots showing significance versus gene expression level in the comparison of L. lactis MG*ΔpepV* with MG1363 or MG*ΔpepVcodY*^R218C^ with MG1363. Genes outside the gray areas have a fold change of ≥2 and *P* value of ≤0.05; genes outside the two dashed lines have a fold change of ≥3 and *P* value of ≤0.01. A sphere around a circle is a measure of the combined expression level of the corresponding gene in MG1363 plus MGΔ*pepV* or in MG1363 plus MGΔ*pepVcodY*^R218C^. (C) (Left) Heat map showing the fold change in gene expression in MG*ΔpepV* or MGΔ*pepVcodY*^R218C^ of CodY regulon members, each strain in a comparison with MG1363. The number inside each rectangle is the fold change. NA, no significant difference. (Right) Schematic representation of corresponding CodY regulon genes/operons. Genes are drawn to scale; their coloring corresponds to the heat map color key of MGΔ*pepVcodY*^R218C^ versus MG1363. Gray, no significant change.

Figure 6A gives the absolute numbers of significantly up- and downregulated genes, while Fig. 6B shows the distribution of affected genes for each comparison. As can be seen from Fig. 6B, the extent of transcriptome changes in MG*ΔpepVcodY*^R218^ (log fold change [FC] from −5 to 7) is larger than in MG*ΔpepV* (log FC from −4 to 3). The heat map of high-fold-change top hits (Fig. S2) shows similar patterns for MG*ΔpepV* and MG*ΔpepVcodY*^R218^. This implies that although the magnitude of the response of MG*ΔpepVcodY*^R218^ is larger than that of MG*ΔpepV*, the mechanisms by which these two mutants react to SMGG are probably the same. As is clear from the analysis presented in Fig. 6B, several of the genes that are part of the CodY regulon of L. lactis (6) are upregulated in MG*ΔpepV* upon 30 min of SMGG stress. As expected, the CodY regulon is strongly depressed in MG*ΔpepVcodY*^R218^. Apparently, (some of) the CodY regulon members allow MG*ΔpepVcodY*^R218^ to regrow faster than MG*ΔpepV* in the presence of glycine.

The CodY regulon of L. lactis and other Gram-positive bacteria has been examined thoroughly by several transcriptome and electrophoretic mobility shift assay (EMSA) studies (6, 14, 15, 18, 20, 22–25). As can be seen in Fig. 6B, the majority of the CodY regulon genes are upregulated in both MG*ΔpepV* and MG*ΔpepVcodY*^R218^. As illustrated in Fig. 6C and D, the CodY regulon genes of MG*ΔpepVcodY*^R218C^ can be categorized into two groups: one encompassing those that encode transporters (*opp*, *ctrA* [*bcaP*], and *dppA*) and the other containing genes/operons for biosynthesis of amino acids such as branched-chain amino acids (BCAAs; leucine, isoleucine, and valine), histidine, glutamate, etc. The dipeptide transporter gene *dppA* is highly upregulated in MG*ΔpepVcodY*^R218C^ but unchanged in MG*ΔpepV*. Increased transport by DppA in MG*ΔpepVcodY*^R218C^ might lead to more dipeptide uptake as an alternative source of alanine. The expression levels of *gltB* and *gltD* are also dramatically increased (35-fold) in MG*ΔpepVcodY*^R218C^ compared to those in MG*ΔpepV* (2-fold). The oligopeptide permease operon (*oppDFBCA*) is also upregulated in both strains under SMGG stress. This could lead to the import of more oligopeptides as potential alanine sources through the action of other (amino)peptidases. Other amino acid synthesis (BCAAs, Asp, and Ser) and transport (*ctrA* [*bcaP*]) genes are also very highly upregulated, although this might be due to the fact that they are the most repressed genes when CodY functions normally (6).

## DISCUSSION

Proper cell wall synthesis is crucial for bacteria in order to maintain cell shape and integrity, to allow proper cell division, and to resist external stresses and internal turgor pressure. Disruption of any component in the process could potentially cause growth inhibition or even cell death (26, 27). Gram-positive bacteria have thick cell walls made up of peptidoglycan (PG) polymers. In L. lactis, both l-Ala and d-Ala are essential elements of the PG precursor (Fig. 1): l-Ala is coupled to UDP-MurNAc by Mur ligase MurC, while a d-Ala–d-Ala dimer is added by MurF to the ends of the pentapeptides that form the bridges between two PG strands (9). The amino acid glycine can weaken the cell wall by replacing alanine, which disrupts the synthesis process. MurC is inhibited by glycine, causing an accumulation of UDP-MurNAc, while the cross bridge links cannot be formed when d-Ala is replaced by glycine at position four in the pentapeptide (16, 17). In other words, a sufficient alanine pool inside the cell is very important or may be even essential for cell wall synthesis in L. lactis.

The intracellular dipeptidase PepV of L. lactis MG1363 hydrolyzes a broad range of dipeptides, among which are some containing an alanine residue(s) (8). PepV of Lactobacillus delbrueckii is a relatively nonspecific dipeptidase but has a notable high activity when an N-terminal d-Ala residue is present (28). From the crystal structure of PepV from L. delbrueckii, it was concluded that the enzyme preferentially should use dipeptides with a large hydrophobic side chain at the N terminus (29). The prolonged lag phase of MG*ΔpepV* in a rich medium with a high concentration of glycine can be almost completely restored by the addition of alanine. This suggested that a shortage of intracellular alanine in MGΔ*pepV* leads to glycine toxicity through abnormal PG precursor formation, causing the observed cell shape defects.

Both MGΔ*pepV* and MGΔ*pepVcodY*^R218C^, when resuspended in liquid SMGG, initially showed a decrease in the OD (Fig. 5A), which is suggestive of cell lysis (also clear in Movies S2 and S3). The cell shape defects and long chains observed upon subsequent regrowth suggest improper cell wall synthesis and cell separation (Fig. 5B). A Δ*dltA* mutant of Streptococcus agalactiae showed a 20-fold decrease in surface rigidity compared to that of the wild type, which could be recovered by the complementation of *dltA*. This suggests that d-Ala is very important for cell rigidity (30). Two possibilities have been proposed to explain L. lactis cell lysis through d-Ala depletion. Alteration of PG makes it more susceptible to the autolysin AcmA, or the reductive d-alanylation of lipoteichoic acid results in a decrease of AcmA degradation, increasing lysis activity (31). The vital role that d-Ala plays in the proper functioning of PG might also be due to its role in cross bridge formation. Indeed, depletion of d-Asp in the PG cross bridge in L. lactis affects cell integrity, resulting in cell shape defects. A shortage of aspartate, the source of the third amino acid of the pentapeptide, mDAP, limits PG synthesis in Bacillus subtilis (32, 33).

The fact that MG*ΔpepVcodY*^R218C^ grows better than MG*ΔpepV* should be linked to the mutation in CodY. Transcriptome sequencing (RNA-seq), comparing MGΔ*pepVcodY*^R218C^ and MGΔ*pepV* with the wild type, was used to try to uncover the underlying mechanism. The oligopeptide transporter Opp is upregulated in both mutants, while the peptide transporters DppA and DtpT are upregulated only in MG*ΔpepVcodY*^R218C^. An increase in the latter two transporters could result in the import of more di- and tripeptides and thus contribute to the intracellular alanine pool. Alanine can also be synthesized via glutamate and pyruvate (Fig. 1). An increase in the intracellular glutamate pool through increased uptake and/or biosynthesis might lead to supplementation of the alanine pool via the alanine-synthesizing transaminase AspC (11). Although *aspC* expression is not upregulated in either strain relative to MG1363, this need not be required if the wild-type level of AspC is already enough to deal with the increased glutamate pool.

**FIG 7:**
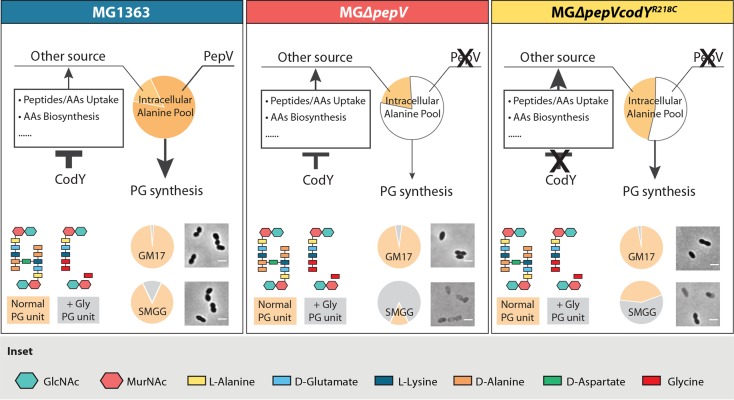
Model showing the response to glycine of MG1363, MG*ΔpepV*, and MG*ΔpepVcodY^R218C^*. Relative contributions of intracellular alanine pools derived from PepV activity and from other sources are shown in the topmost pie charts in each panel. White, no contribution. Differences in the thicknesses of arrows and T-symbols show the relative degrees of contribution and inhibition, respectively. The cross indicates the deletion of *pepV* or the functional loss of CodY. Typical cell morphologies of the strains are shown in the bottom right of each panel. White bars, 2 μm. The composition of the peptidoglycan (PG) unit when cells were grown in GM17 or SMGG is shown at the bottom left of each panel. Relative ratios of normal and abnormal PG in GM17 and SMGG are shown by the small pie charts next to the microscopy images.

The precise mechanism by which PepV affects PG synthesis is unknown. We propose a model (Fig. 7) in which PepV is the main contributor to the intracellular alanine pool in the rich M17 medium. The pentapeptide of PG of wild-type MG1363 predominantly contains alanine in positions 1 (l-Ala) and 4/5 (d-Ala). In MGΔ*pepV*, because it is more resistant to vancomycin than MG1363, (some of) the d-Ala might be replaced by its analogues (data not shown). When a high concentration of glycine is introduced, some of the alanine in PG will be replaced by glycine in the wild-type strain, affecting the PG precursor, but not to the extent that we can observe by light microscopy or as a decrease in OD. As for MGΔ*pepV*, the replacement of alanine is significantly higher than in the wild type, which ultimately leads to cell shape defects and a lowering of the OD. In MGΔ*pepV*CodY^R218C^, a rescue mechanism is activated: through the functional loss of the global repressor CodY, genes related to (oligo)peptide transport and alanine biosynthesis are significantly upregulated, alleviating the alanine shortage and finally decreasing the restoration period (Fig. 7).

## MATERIALS AND METHODS

### Bacterial strains, media, and culture conditions.

Bacterial strains used in this study are listed in Table 1. L. lactis MG1363 and its derivatives were grown at 30°C in Difco M17 medium (BD, Franklin Lakes, NJ) containing 0.5% (wt/vol) glucose (GM17). When required, erythromycin was added at a final concentration of 5 μg ml^−1^. Chemically defined SA medium with 0.5% (wt/vol) glucose and 20 μg ml^−1^ of 5-fluoroorotic acid (5-FOA; Sigma-Aldrich, St. Louis, MO) as a sole pyrimidine source was used for the generation of chromosomal knockouts (34). Escherichia coli DH5α was used for cloning purposes; it was grown aerobically at 37°C in LB medium (Formedium, Norfolk, UK) with, when required, erythromycin at a final concentration of 200 μg ml^−1^. All chemicals were obtained from Sigma-Aldrich. Unless stated otherwise, for glycine inhibition studies, 0.5 M sucrose and 1.5% (wt/vol) glycine were added into GM17 (SMGG); for alanine addition, 240 mg ml^−1^ of l-Ala–d-Ala or 120 mg ml^−1^ of l-Ala–l-Ala was added.

**TABLE 1:** Strains and plasmids used in this study

Strain or plasmid	Description	Reference or source
E. coli strain		
DH5α	F^−^ Φ80*lacZ*ΔM15 Δ(*lacZYA-argF)U169 rec1A end1A hsdR17 gyrA96 supE44 thi-1 relA1*	43
L. lactis strains		
MG1363	L. lactis subsp. *cremoris* plasmid-free derivative of NCDO712	44
MG*ΔpepV*	MG1363 carrying a chromosomal deletion of *pepV*	This study
MG*ΔpepVcodY*^R218C^	MG*ΔpepV* with mutation in CodY 218 residue from arginine to cysteine	This study
Plasmids		
pCS1966	Integration vector for L. lactis	34
pCS1966-pepV	pCS1966 containing *pepV* deletion construct	This study

### Recombinant DNA techniques and oligonucleotides.

Standard molecular cloning techniques were performed essentially as described previously (26). Chromosomal DNA from L. lactis was isolated using the GenElute genomic DNA kit (Sigma-Aldrich, St. Louis, MO). Plasmids and PCR products were isolated and purified using the High Pure plasmid isolation and PCR purification kit (Roche Applied Science, Mannheim, Germany) and the NucleoSpin gel and PCR cleanup kit (Macherey-Nagel, Düren, Germany) according to the manufacturer’s instructions. PCRs were performed with Phusion or DreamTaq polymerase (both from Fermentas) according to the manufacturer’s protocol. The obtained PCR fragments were mixed and treated with the Quick-Fusion enzyme mixture (BIO-Connect Services BV), yielding 15-nucleotide overhangs annealing to complementary overhangs. No ligation was required, Quick-Fusion-treated mixtures were directly used to transform E. coli. Oligonucleotides employed in this study are listed in Table 2 and were purchased from Biolegio BV (Nijmegen, the Netherlands). Competent E. coli cells were transformed using heat shock, while electrocompetent L. lactis cells were transformed using electroporation with a Bio-Rad gene pulser (Bio-Rad Laboratories, Richmond, CA). All nucleotide sequencing was performed at Macrogen Europe (Amsterdam, the Netherlands).

**TABLE 2:** Sequences of oligonucleotides used for plasmid and strain construction

Primer name	Sequence (5′ → 3′)
pCS1966_1FW	GTGCCTAATGAGTGAGCTAACTC
pCS1966_1RV	GTGGAATTGTGAGCGGATAAC
33-V_UP_FW	CGCTCACAATTCCACCGAGAAATAGACTTAGCGTT
34-V_UP_RV	TCGCTTGGTTGTATAACCATCACGTTCG
35-V_DOWN_FW	TATACAACCAAGCGAATGAAATGAAACCT
36-V_DOWN_RV	TCACTCATTAGGCACTTAGTCACCAGATGATTTCGT
87-Seq_mid_05_V	GTACTTTTCTAGCTCCATTGTTG
0099-Seq_F_pCS1966	CTGCAGGAATTCGATATCAAGC
0100-Seq_R_pCS1966	CTTTGAGTGAGCTGATACCGC

### Construction of the L. lactis deletion strain MG*ΔpepV*.

All plasmids and strains that were used or constructed during this study are listed in Table 1. Nucleotide sequences of the primers are presented in Table 2. Pertinent regions of all plasmids were sequenced to confirm their proper nucleotide sequences. The flanking regions of *pepV* were amplified using 33-V_UP_FW/34-V_UP_RV and 35-V_DOWN_FW/36-V_DOWN_RV, while the linearized vector was amplified by pCS1966_1FW/pCS1966_1RV. The fragments were then fused with the Quick-Fusion cloning kit (BiMake; catalog no. B22612) according to the manufacturer’s instructions, with the exception of using only one-half of the recommended volume per reaction. Each reaction was directly used to transform competent E. coli. The resulting vector was designated pCS1966-pepV. Vector pCS1966-pepV was introduced into L. lactis MG1363 via electroporation (27); cells in which the two-step homologous recombination event had occurred were selected by growing them on selective SA medium plates supplemented with 20 g ml^−1^ of 5-fluoroorotic acid hydrate (34). The obtained strain was labeled MG*ΔpepV*. The chromosomal structure of the deletion strain was confirmed by PCR analysis and sequencing.

### OD measurements using microtiter plate reader.

L. lactis cells were grown overnight in GM17 and then inoculated to a starting optical density (OD) of 0.05 in SMGG and divided as triplicates in a transparent 96-well microtiter plate. OD at 600 nm (OD_600_) was measured every 10 min at 30°C in an Infinite 200 Pro plate reader (Tecan Group Ltd., Männedorf, Switzerland) with I-control 1.10.4.0 software (Tecan Group Ltd.).

### Microscopy for time points, LIVE/DEAD cell staining, and time-lapse.

All micrographs were obtained with a DeltaVision Elite inverted epifluorescence microscope (Applied Precision, GE Healthcare, Issaquah, WA) equipped with a stage holder, a climate chamber, a seven-color combined set InsightSSI solid-state illumination module, and an scientific complementary metal oxide semiconductor (sCMOS) camera (PCO AG, Kelheim, Germany). A 100× phase-contrast objective (numerical aperture [NA] 1.4, oil immersion, DV) was used for image capturing, in combination with SoftWorX 3.6.0 software (Applied Precision) to control the microscope setup. For time point microscopy, a standard microscope slide was prepared with a layer of solidified agarose (1.5%, wt/vol, in phosphate-buffered saline [PBS]), and 1 μl of bacterial cells was spotted onto the agar. The sample was covered with a standard microscope coverslip for microscopic observations. For LIVE/DEAD cell staining, cell treated using a LIVE/DEAD BacLight bacterial viability kit according to the manufacturer’s protocol. To prevent phototoxicity, the excitation light (480 to 500 nm for 0.1 s for SYTO9 and 541 to 569 nm for 0.3 s for propidium iodide) was limited to 10% of the output of a 100-W Hg vapor lamp by neutral density filters. Emission wavelengths were 509 to 547 nm (SYTO9) and 580 to 653 nm (propidium iodide). For Movies S1, S2, and S3, microscope slides were incubated in the temperature-controlled (cube and box incubation system; Life Imaging Services) automated microscope (DeltaVision Elite) at 30°C for up to 11 days. Images were obtained every 10 min, the XYZ position stored in the microscope control software SoftWorX.

### Flow cytometry.

L. lactis cells were grown overnight in GM17 as described above. Overnight cultures were inoculated in GM17 at an OD of 0.05 and incubated at 30°C. When the OD reached 0.7, cells were spun down and washed in PBS. A total of 150 μl of cell culture was resuspended in 2 ml of PBS. A constant volume of 5 μl was analyzed by flow cytometry (flow rate at 10 μl/min and collection at 30 s) to calculate the number of cells in each culture. Raw data were collected using FACS Diva software (BD Biosciences), and FlowJo software was used for data analysis.

### Genome sequencing and data analysis.

For genome sequencing, a single colony was grown in 4 ml of GM17 broth at 30°C. Overnight cultures were diluted 50-fold in fresh GM17 broth and grown until the late exponential growth phase. Cells were collected by centrifugation at 10,000 rpm for 2 min, and total DNA was isolated with a GenElute bacterial genomic DNA kit (Sigma-Aldrich) according to the manufacturer’s protocol. The genomes were determined at GATC Biotech (Germany) with an Illumina HiSeq sequencing system. A total of 5 million paired reads (150 bp) were generated. FastQC version 0.11.5 (https://www.bioinformatics.babraham.ac.uk/projects/fastqc/) was used to examine the quality of the reads; low-quality reads were removed with Trimmomatic version 0.38 (27). The reads were assembled *de novo* using SPAdes version 3.11.1 with default parameters (35). At the assembly stage, sequence reads were aligned to the previously assembled L. lactis MG1363 genome sequence (NCBI accession number NC_009004). Breseq (36) was used to determine point mutations compared to MG1363.

### RNA isolation and RNA sequencing.

All procedures were executed at 4°C unless otherwise stated, and all solutions were diethyl pyrocarbonate (DEPC) treated and subsequently autoclaved. Frozen cell pellets were resuspended in 400 μl of TE buffer (10 mM Tris-HCl, 1 mM EDTA [pH 7.4]) and added to 50 μl of 10% sodium dodecyl sulfate (SDS), 500 μl of phenol-chloroform (1:1, vol/vol), and 0.5 g of glass beads (75 to 150 μm; Thermo Fisher Scientific, Rockford, IL). The cells were disrupted by shaking 2 times for 45 s in a Biospec Mini-BeadBeater (Biospec Products, Bartlesville, OK), with cooling on ice for 1 min between the shaking steps. Subsequently, the cell suspension was centrifuged at 14,000 rpm for 10 min. The upper phase containing the nucleic acids was treated with 500 μl of chloroform and centrifuged as described above. Nucleic acids in the water phase were precipitated with sodium acetate and ethanol. The nucleic acid pellet was resuspended in 100 μl of buffer consisting of 82 μl of Milli-Q water, 10 μl of 10× DNase I buffer, 5 μl of RNase-free DNase I (Roche Diagnostics GmbH, Mannheim, Germany), and 3 μl of RiboLock RNase inhibitor (Fermentas/Thermo Scientific, Vilnius, Lithuania) and treated for 30 min at 37°C. The RNA was then purified using standard phenol-chloroform extraction and sodium acetate and ethanol precipitation. RNA pellets were resuspended in 50 μl of elution buffer from the High Pure RNA isolation kit (Roche Diagnostics, Almere, the Netherlands) and stored at −80°C. RNA concentration was measured with a NanoDrop ND-1000 (Thermo Fisher Scientific). As a measure of RNA quality, the integrity of the 16S/23S rRNA and the presence of any DNA contamination were assessed by using an Agilent 2100 Bioanalyzer (Agilent Technologies, Waldbronn, Germany). cDNA library preparation and RNA sequencing were performed by BGI Genomics Corporation (Copenhagen, Denmark).

### RNA-seq data analysis.

Raw sequence reads were analyzed for quality and trimmed with a PHRED score of >28. Read alignment was performed on the genomic DNA of *L. lactic* MG1363 using Bowtie 2 (37). Values for reads per kilobase per million reads (RPKM) were used as an input for the T-REx analysis pipeline (21) together with a text file describing the factors, contrasts, and classes. T-Rex, which employs EdgeR, was used to perform all statistical analyses (21).

## Supplementary Material

Supplemental file 1

Supplemental file 2

Supplemental file 3

Supplemental file 4
