# Supplementary material for: Lysis of a Lactococcus lactis Dipeptidase Mutant and Rescue by Mutation in the Pleiotropic Regulator CodY
Source: Appl Environ Microbiol. 2020 Apr 1;86(8):e02937-19. doi: 10.1128/AEM.02937-19 (PMC7117943; doi:10.1128/AEM.02937-19)
Supplement: Supplemental file 1 [file AEM.02937-19-s0001.pdf]

Supplemental Figures

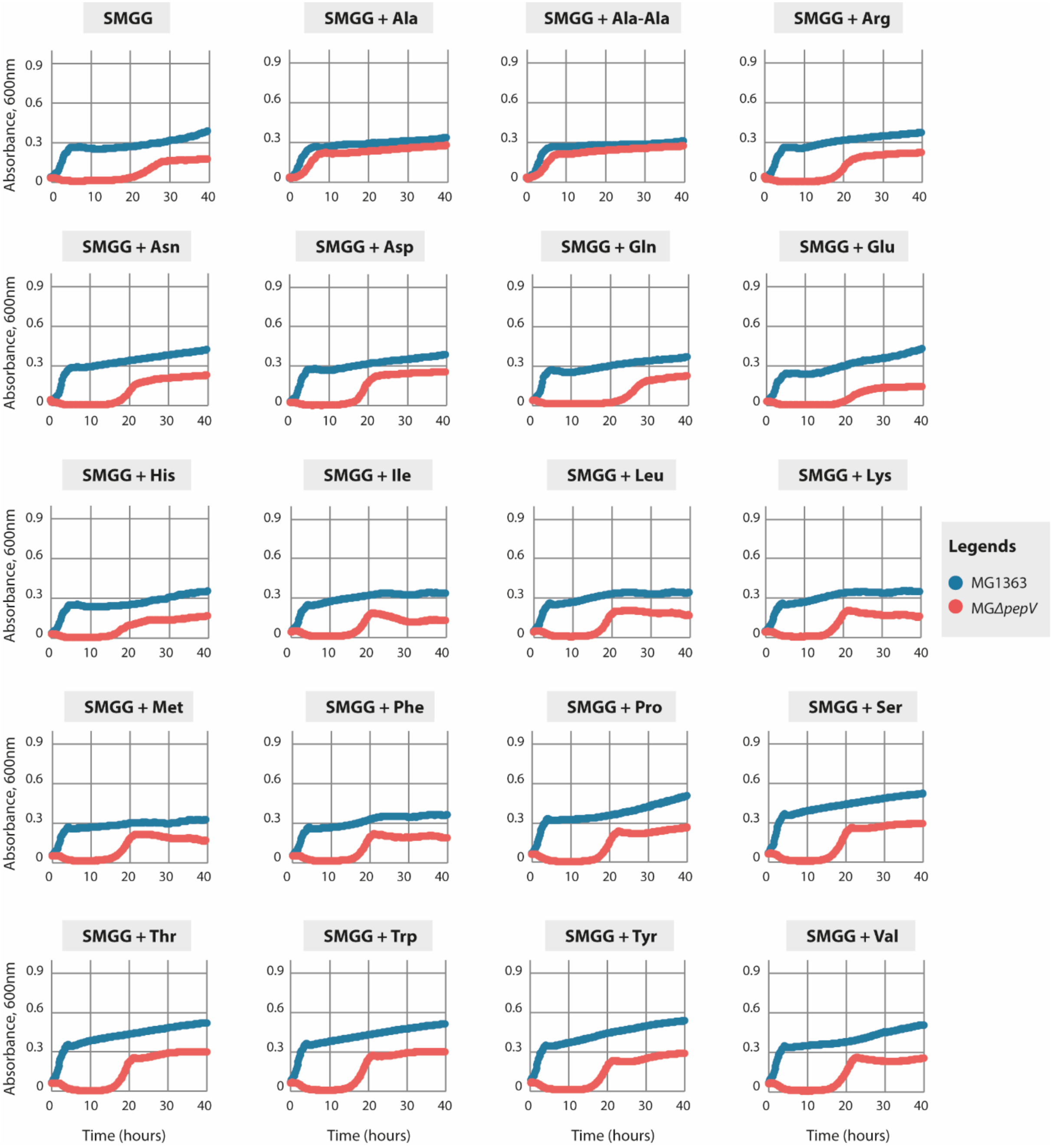

Supplemental Figure 1 Growth curves of *L. lactis* MG1363 and MG*ΔpepV* in SMGG with 18 amino acids or the dipeptide Ala-Ala.

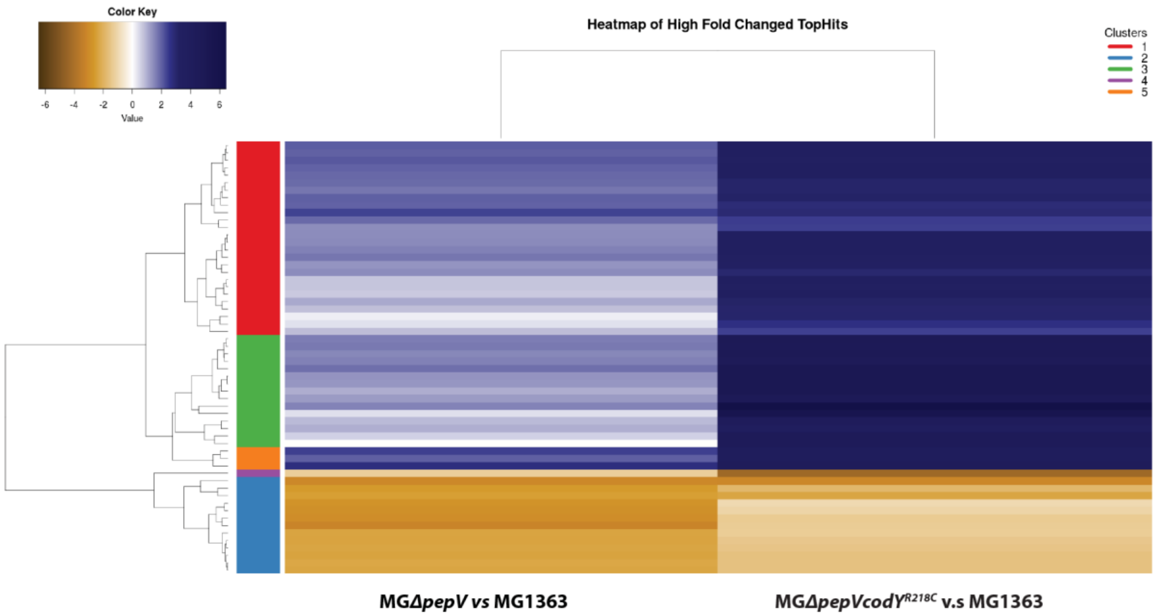

Supplemental Figure 2 Heatmap showing the difference in high fold-changed genes between each mutant, MG*ΔpepV* and MG*ΔpepVcodY*^{R218C}, and the wildtype. Genes that did not show statistically significant changes in transcript levels ($p>0.05$) or fold-change less than two (FC<2) were excluded. The differentially expressed genes ($p<0.05$) between mutants and WT libraries were normalized, centered and automatically clustered by web server T-Rex. Brown: lower expression; blue: higher expression.

Suppl Movie 1-3: Time-lapse movie of three strains (1. MG1363 2. MGΔpepV 3. MG*ΔpepVcodY*^{R218C}) incubating on SMGG-agar slides. Overnight culture of three strains were inoculated in GM17 at starting OD 0.05, when the cultures reached the mid-log phase (around OD 0.6), the cells were spun down and resuspended SMGG. 1 μl of cell culture was spotted on the SMGG-agar slide. Every second in the videos corresponds to 2 hours in reality.
